# Concomitant Temporomandibular Joint Replacement and Orthognathic Surgery

**DOI:** 10.3390/diagnostics13152486

**Published:** 2023-07-26

**Authors:** Felix Jose Amarista, Daniel E. Perez

**Affiliations:** Department of Oral and Maxillofacial Surgery, University of Texas Health Science Center at San Antonio, 8210 Floyd Curl Drive, Mail Code 8124, San Antonio, TX 78229, USA; perezd5@uthscsa.edu

**Keywords:** temporomandibular joint disorders, dentofacial deformities, alloplastic TMJ reconstruction, orthognathic surgery

## Abstract

The treatment of patients with severe temporomandibular joint (TMJ) disorders and associated skeletal discrepancies presents a complex challenge for oral and maxillofacial surgeons. It is widely recognized that TMDs can impact the stability and outcomes of surgical treatments for dentofacial deformities. Consequently, addressing TMDs prior to or in conjunction with orthodontic or surgical interventions may be necessary to achieve optimal and long-lasting results. Alloplastic temporomandibular joint replacement (TMJR) and orthognathic surgery have emerged as the standard approach due to their predictability, long-term stability and excellent outcomes when addressing end-stage TMJ disease in conjunction with DFDs as it provides a comprehensive solution to address both functional and aesthetic aspects of these patients’ conditions. Understanding the appropriate utilization of TMJR in conjunction with orthognathic surgery can lead to improved treatment planning and successful outcomes for patients with complex TMJ disorders and associated dentofacial deformities. This review aims to discuss the indications, preoperative evaluation, staging, sequencing, and surgical considerations involved in utilizing alloplastic TMJ replacement in the presence of dentofacial deformities.

## 1. Introduction

Temporomandibular joint disorders (TMDs) involve a range of conditions that can cause pain and dysfunction in the joint and muscles of mastication. The relationship between TMDs and dentofacial deformities (DFDs) remains controversial in the literature. Some authors argue that there is a strong association between malocclusion and some types of deformities (Class II malocclusion, hyperdivergent growth patterns, asymmetries, and open bites) and the development of TMJ disorders [1,2]. However, others suggest that the TMJ pathology may be the causative factor for the dentofacial deformity [3,4].

It is widely recognized that TMDs can impact the stability and outcomes of surgical treatments for dentofacial deformities. Consequently, addressing TMDs prior to or in conjunction with orthodontic or surgical interventions may be necessary to achieve optimal and long-lasting results [5,6,7,8,9]. Failure to address TMJ pathology can lead to the persistence or worsening of pre-surgical symptoms and the original deformity, accompanied by deteriorating occlusion, jaw dysfunction, facial imbalance, and increased pain. Mild forms of TMDs in patients with DFDs can often be managed conservatively (e.g., physical therapy, orthotics, etc.). Surgical treatment options may include arthrocentesis, arthroscopic lysis and lavage, and orthognathic surgery following resolution of the TMJ pathology. However, advanced stages of TMJ disease accompanied by dentofacial deformities may require TMJ intervention. Alloplastic TMJ reconstruction (TMJR) has emerged as the standard approach due to its predictability, long-term stability and excellent outcomes when addressing end-stage TMJ disease in conjunction with DFDs [10,11,12,13].

The aim of the present review is to discuss indications, preoperative evaluation, staging, sequencing and surgical pearls when employing alloplastic TMJ replacement in the presence of dentofacial deformities.

## 2. Indications for TMJR

Alloplastic TMJ reconstruction is typically recommended for individuals with advanced stages of TMJ pathology. The literature provides several indications for TMJR, including conditions characterized by condylar bone loss, degenerative joint disease (such as osteoarthritis or osteoarthrosis), high inflammatory joint diseases (such as rheumatoid arthritis, ankylosing spondylitis or psoriatic arthritis), TMJ ankylosis, congenital or acquired agenesis or hypoplasia of the TMJ, previous prosthetic reconstruction that has failed, patients with unsatisfactory postoperative outcomes following costochondral grafting or those who have undergone multiple prior surgical TMJ procedures [14,15,16,17,18].

**Figure 1:**
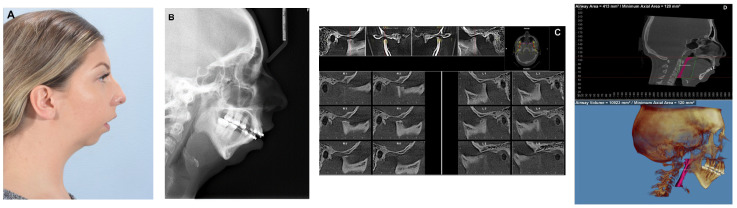
(**A**) 24-year-old female with bilateral condylar resorption, high occlusal and mandibular plane angles, retruded mandible, severe Class II appearance and anterior open bite. (**B**) Lateral cephalogram confirming clinical findings. (**C**) TMJ views from CBCT confirm severe condylar resorption. (**D**) Airway analysis shows severely decreased airway space (Airway volume = 10,923 mm^3^, Minimum Axial Area = 120 mm^2^).

When assessing a patient who requires TMJ reconstruction, it is crucial to consider that approximately two-thirds of these patients can experience significant advantages by undergoing concurrent orthognathic surgery [11]. This decision is made following a comprehensive clinical examination, which includes a thorough assessment of the patient’s complaints, expectations and clinical exam including dental models or intraoral dental scans, as well as the utilization of radiographic aids (panoramic Xray, lateral cephalogram and cone-beam computed tomography (CBCT) or computerized tomography (CT) scan) to gather additional information. End-stage TMJ pathology, particularly when condylar degeneration is present, can lead to loss of posterior vertical height and high occlusal plane angles, Class II malocclusions, anterior open bite and decreased airway space (Figure 1).

For these patients, the combined approach of TMJR with orthognathic surgery not only improves functional aspects (better chewing and opening) but also enhances airway and breathing capabilities, produces superior aesthetic outcomes and can lead to a reduction or even elimination of pain [11].

The young age of the patient represents a relative contraindication for utilizing an alloplastic TMJ device. This is primarily due to the inert nature of total alloplastic TMJ reconstruction prostheses, which lack the potential for growth. Thus, before considering their use in growing patients as an alternative to autogenous tissue, a careful evaluation of the benefits is imperative. Nevertheless, certain authors recognize the predictability and favorable outcomes associated with this approach in growing patients, considering it a valuable modality in specific cases [19,20,21]. Today, most patients over the age of 12, once the second molars erupted, can be considered candidates for TMJ alloplastic reconstruction.

Once considering the utilization of an alloplastic device, it is of utmost importance for the surgeon to engage in a thorough discussion with the patient regarding the outcomes of the procedure. This discussion should be based on the existing literature, which encompasses various aspects, including the longevity and durability of the devices involved as well as the potential complications including nerve damage (CNV and CNVII) and infection risks.

## 3. Device Selection (Stock vs. Custom)

Alloplastic reconstruction of the TMJ can be performed using either a stock or a custom-made device, each with its own set of advantages and disadvantages. There are only two Food and Drug Administration—approved TMJR systems available in the US: the Stryker-TMJ Concepts patient-fitted joint prosthesis (Ventura, CA, USA) and the Zimmer Biomet stock prosthesis (Jacksonville, FL, USA).

Stock devices are typically readily available, but they come with limitations in terms of sizes and shapes. One of their main advantages is reduced cost and the possibility of utilizing all titanium components for the allergy-confirmed individual. With stock prostheses, the patient’s anatomy needs to be adapted to the prosthesis, which may pose challenges. It is important to note that stock prostheses are not recommended or approved for mandibular advancement procedures. On the other hand, custom-made devices are specifically tailored to fit the patient’s unique anatomy. They offer the advantage of being able to address severe dentofacial deformities that require significant mandibular advancements and the presence of a posterior stop in the glenoid fossa component to prevent dislocation of the condylar component. However, one drawback of custom devices is that they typically require longer waiting times for the fabrication of the prosthesis, which may extend the overall treatment timeline.

In a study published by our institution (Brown et al. [22]), with the aim of evaluating whether or not a stock prosthesis could have been used to reconstruct a TMJ previously reconstructed with a custom device, we found that 40% of the reconstructed joints had concomitant orthognathic surgery and of those, 60% could have used a stock prosthesis. However, most of the patients who might have been able to be treated with a stock prosthesis did not have significant maxillary compensations to correct and underwent minimal mandibular advancements. On the other hand, with significant mandibular advancements, the angle of the stock prosthesis becomes acute and increases the risk of posterior dislocation since the stock glenoid fossa component does not have a posterior stop to prevent posterior dislocation of the condylar component. Additionally, in patients with congenital disorders, a stock prosthesis could not be properly adapted to fit the altered anatomy.

A precise diagnosis and thorough preoperative planning are crucial to determine the most appropriate system for achieving optimal outcomes in TMJ reconstruction. While there are situations where a stock device can be utilized in combination with orthognathic surgery, it is important to note that alloplastic TMJ reconstruction is a technique-sensitive procedure, and the experience of the surgeon plays a significant role in the final results.

Considering these factors, we strongly recommend the use of a custom-made device in cases involving concomitant orthognathic surgery. Custom devices, tailored to the patient’s specific anatomy, can provide a more precise fit and facilitate the correction of dentofacial deformities with greater accuracy and speed [10,23]. This approach helps to optimize the overall outcome of the procedure. The planning phase resembles that of conventional virtual planning for orthognathic surgery with the caveat that the mandible is moved using a TMJR and not with a bilateral sagittal split osteotomy (BSSO) or inverted L osteotomy.

## 4. Preoperative Planning and Virtual Surgical Planning

Once the decision is made to proceed with TMJR and concomitant orthognathic surgery, a comprehensive clinical examination is conducted in order to gather all clinical information, encompassing the collection of dental models or intraoral scans.

The treatment planning process for concomitant TMJ total joint replacement cases involves a comprehensive approach that incorporates various factors to achieve an optimal treatment outcome. This process relies on cephalometric analysis, predictions, clinical evaluation to obtain midlines, maxillary or mandibular cant, evaluation of asymmetries and tooth-to-lip relationship as well as dental models, which serve as essential tools in guiding the movement of the upper and lower jaws. By utilizing these techniques, the treatment plan aims to establish a harmonious balance between function, facial aesthetics, occlusion and oropharyngeal airway dimensions. Additionally, if the patient has a diagnosis of obstructive sleep apnea (OSA), polysomnography should be considered.

The traditional planning involving dental cast and model surgery is no longer used. Virtual surgical planning (VSP) has proven to be a reliable method to preoperatively study and plan surgery. It additionally provides accuracy and precision in surgery when used with a custom-made TMJ replacement prosthesis [24].

A medical-grade CT scan with 1 mm cuts is obtained and the resulting Digital Imaging and Communications in Medicine (DICOM) files from the CT scan are forwarded to the planning company. We prefer the accuracy of a medical-grade CT, however, high-resolution CBCT can be utilized as well. A VSP session is performed to determine the final positioning of the maxilla and mandible as customary in any bimaxillary orthognathic surgery case.

There are multiple sequences available for performing the virtual surgical planning (VSP) session. The following sequence is the authors’ preference:

1. Verify the final occlusion: Obtained from scanning the final occlusion after conventional model surgery or via the virtual setup of the occlusion. Today, we favor occlusions that are set by the engineer and revised by the surgeon during the session.
2. Orient the head: Verify the midline and cant with the engineer and correlate them with the clinical examination to ensure proper alignment.
3. Correct midlines and maxillary cant: Make any necessary adjustments to achieve proper alignment of the midlines and correct any maxillary cant issues. Here, the clinical impression is always the most important.
4. Perform maxillary incisor movement: Make anterior–posterior, vertical and transverse adjustments as needed to achieve the desired positioning of the maxillary incisors.
5. Check the final profile and modify the occlusal plane: Evaluate the profile and make any necessary modifications to the occlusal plane. Patients with advanced TMJ diseases typically present steep occlusal and/or mandibular plane angles and will often require significant counterclockwise rotation of the maxillo-mandibular complex.
6. Consider genioplasty: Assess the need for genioplasty and perform it if required to achieve the desired facial profile and aesthetic outcomes.
7. Adjust the proximal segment virtually (only if unilateral TMJR and contralateral sagittal split osteotomy (SSO)). Make any necessary adjustments to achieve proper alignment and positioning of the proximal segment.
8. Correct yaw: Make corrections to the yaw alignment, if necessary, by viewing from caudal aspect of jaws.
9. Verify the condylectomy virtual cuts to allow for proper space for the TMJR and decide if a coronoidotomy is needed.
10. Check intermediate occlusion for fabrication of an intermediate splint: Depending on the chosen sequence (mandible vs. maxilla first), evaluate the intermediate occlusion to determine the fabrication of an intermediate splint. Typically, a mandible first approach is used, and an intermediate splint is made in that position.

It is important to note that this sequence reflects the author’s preference, and individual cases may require modifications based on specific patient needs and clinical judgment.

**Figure 2:**
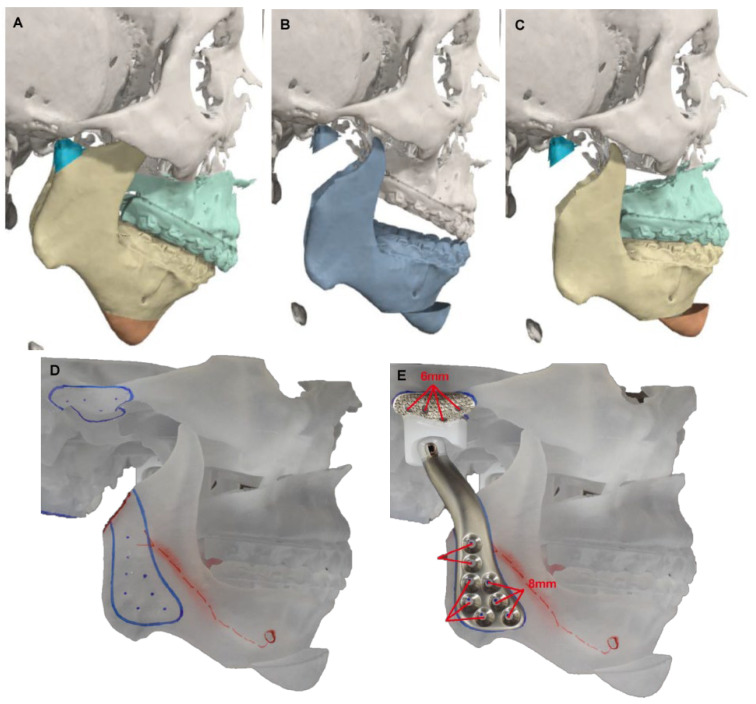
Steps for fabrication of a custom alloplastic TMJ prosthesis. (**A**) Virtual preoperative position. (**B**) Virtual intermediate position with mandible first approach. Note the posterior open bite and virtual condylectomy cuts to allow adequate space for the TMJR. (**C**) Virtual final position producing acceptable facial profile. (**D**) Printed STL model with initial design of custom alloplastic TMJ prosthesis. (**E**) Custom alloplastic TMJ prosthesis device with manufacturer recommendations for screw length.

Once the optimal position is established through VSP, the next step involves the transfer of the stereolithography (STL) files obtained from the VSP to the manufacturer of the alloplastic TMJ device (Figure 2). The manufacturer will then design the prosthesis. In cases where there are significant irregularities in the mandibular ramus, the authors recommend reducing them before fabricating the prosthesis. The goal is to remove any interferences and improve the adaptation of the prosthesis to the mandibular ramus. Additionally, it will allow the surgeon to manipulate the mandibular component up-down during surgery in order to make sure that the mandibular component is fully seated in the glenoid fossa component.

Given the extended fabrication duration of TMJ devices and the possibility of some dental movement during this time, the authors prefer not to fabricate the intermediate or final occlusal splint during the initial virtual surgical planning (VSP) session. Instead, 2–4 weeks before the scheduled procedure, new dental models or intraoral scans are acquired. These files are then sent to the VSP company to merge them with the original plan for fabrication of the splints. This approach allows for a more accurate representation of the patient’s current dental anatomy and optimizes the fit and effectiveness of the occlusal splints.

## 5. Staging

When discussing staging in surgery, it is important to consider several decisions regarding the sequence of events. One option is to perform a single-stage TMJ reconstruction, which involves resection and replacement in a single procedure. Alternatively, a two-stage approach can be chosen, utilizing a spacer. Another decision to consider is whether to delay or stage the orthognathic component of the surgery until after the joint replacement has fully healed and sufficient mobility has been achieved.

In cases where the TMJ anatomy requires minor bone reshaping, most TMJR surgeries combined with orthognathic surgery are typically performed using a one-stage approach. This approach offers several advantages, including cost reduction, a single surgical and anesthetic procedure, and only one hospital admission. However, it is important to note that in situations where postoperative physical therapy is crucial, such as in cases of ankylosis or the syndromic patient, the one-stage approach may not be appropriate [25].

When a Le Fort I osteotomy is performed in conjunction with the resection of TMJ ankylosis, it becomes necessary to delay aggressive postoperative physiotherapy for 8 to 10 weeks. This delay allows for adequate healing of the maxilla but it may result in a reduced maximum interincisal opening (MIO) after surgery. Due to this concern, our institution does not perform cases of ankylosis with a Le Fort I osteotomy simultaneously. Instead, we recommend a two-stage approach: correcting the ankylosis first with final mandibular positioning in the first operation and implementing aggressive physiotherapy, followed by a secondary procedure involving a Le Fort I osteotomy.

A two-stage approach to TMJ reconstruction is recommended in several other scenarios, including the following:

1. Failed previous alloplastic joint replacements requiring significant bone reshaping: In cases where previous TMJ replacements have been unsuccessful and extensive bone reshaping is necessary, a two-stage approach is preferable. This approach allows for adequate healing and preparation before proceeding with the definitive joint replacement surgery.
2. Significant mandibular advancements with a high risk of intraoral mucosal perforation: When significant mandibular advancements are required, there is an increased risk of large perforations in the oral mucosa along the anterior border of the ramus, which can lead to infections. To minimize this risk, a two-stage approach is advisable utilizing a temporary plate with condylar component and delaying the prosthesis placement until the intraoral wound has healed. The initial stage focuses on achieving the desired mandibular advancement while ensuring proper healing and reduced chances of infection.
3. Severe dentofacial deformities and limited maximum interincisal opening (MIO): In cases where patients present with severe dentofacial deformities and limited MIO, orthodontic treatment may be challenging or even impossible until the jaw opening is improved. In such situations, a two-stage approach is recommended. The first stage involves addressing the limited MIO through gap arthroplasties and mandibular TMJR to allow for physical therapy. Once an improved jaw opening is achieved, orthodontic treatment can be initiated and a delayed Le Fort I osteotomy can be conducted to correct the dentofacial deformities in the second stage.

By adopting a two-stage approach in these specific scenarios, better outcomes can be achieved, ensuring successful TMJR and orthognathic surgeries while minimizing complications.

## 6. Sequencing

Several critical factors must be taken into account when deciding on the surgical sequence. Two key considerations are the disparity in sterility between the TMJ surgical field and the intraoral procedures, as well as the availability of multiple instrument sets.

Regardless of the chosen sequence, it is imperative that the TMJ procedure is conducted within a completely sterile field. Cross-contamination poses a significant risk of infection, which has been reported by multiple authors as the most frequent cause of TMJR failure, often necessitating replacement with a new prosthesis [26,27,28,29,30]. Due to the significant risk associated with contamination and subsequent infection, the authors strongly recommend conducting the TMJR portion of the surgery as the initial step, followed by the completion of the orthognathic procedures.

In situations where only one set of instruments is available, our recommendation is to separate a small set of instruments necessary for the application of maxillomandibular fixation (MMF) onto a separate Mayo stand, including cheek retractors, Minnesota retractors, wire drivers, gauze-packer and 24, 26 and 28-gauge wires. In such cases, the recommended sequence of procedures is as follows:

1. The patient’s face, neck, ears, nose, mouth and endotracheal tube are prepped with betadine and draped following standard protocols. Ear canals are packed with cotton pledges.
2. Isolate the mouth using appropriate barriers such as Ioban^®^, Tegaderm^®^, or similar materials to avoid cross-contamination.
3. Perform the extraoral approaches, including endaural/preauricular and retromandibular/submandibular incisions.
4. Perform condylectomy, discectomy, debridement and coronoidotomy/coronoidectomy as needed.
5. Isolate the TMJ surgical field using suitable barriers (Ioban^®^, Tegaderm^®^, etc.) to maintain sterility.
6. Placement of additional surgical drapes over previous ones to avoid cross-contamination.
7. Cut through the oral barrier membrane (e.g., Ioban^®^, Tegaderm^®^, etc.) place the intermediate splint and secure MMF.
8. Remove the additional surgical drapes. The surgeons change gloves and gowns.
9. Isolate the mouth using appropriate barriers such as Ioban^®^, Tegaderm^®^ or similar materials to maintain a sterile field.
10. Proceed with the placement of TMJ prostheses, including the glenoid fossa and mandibular component.
11. Close the extraoral approaches.
12. Check obtained intermediate occlusion to ensure proper alignment and occlusion.
13. Complete maxillary osteotomy and genioplasty. Stabilize the maxilla and chin with appropriate osteosynthesis materials.
14. Check final occlusion.

Performing the orthognathic surgery first followed by the TMJR procedure is an option when two full sets of instruments are available. However, it is important to note that this approach presents challenges in maintaining the sterility of the surgical fields and minimizing the risk of cross-contamination. Due to these concerns, the authors do not recommend this sequence of procedures. If this protocol is chosen, the sequence of procedures is as follows:

1. The patient’s face, neck, ears, nose, mouth and endotracheal tube are prepped with betadine and draped following standard protocols.
2. Perform the maxillary osteotomy.
3. Place the intermediate splint and secure MMF. In this sequence, a maxilla first intermediate splint is necessary, and one must be aware of the difficulty this represents in patients with unstable or degenerated joints. One can elect to use patient specific implants (PSI) here to obviate the need for an intermediate splint. Today, this is our recommendation if this approach is chosen.
4. Stabilize the maxilla with appropriate osteosynthesis materials.
5. Isolate the mouth using appropriate barriers such as Ioban^®^, Tegaderm^®^, or similar materials to avoid cross contamination.
6. The patient’s face, neck, and ears are re-prepped with betadine. Ear canals are packed with cotton pledged.
7. Perform the extraoral approaches, including endaural/preauricular and retromandibular/submandibular incisions.
8. Perform condylectomy, discectomy, debridement and coronoidotomy/coronoidectomy as needed.
9. Isolate the TMJ surgical field using suitable barriers (Ioban^®^, Tegaderm^®^, etc.) to maintain sterility.
10. Placement of additional surgical drapes over previous ones to avoid cross-contamination.
11. Place the final splint and secure MMF.
12. Remove the additional surgical drapes. The surgeons change gloves and gowns.
13. Isolate the mouth using appropriate barriers such as Ioban^®^, Tegaderm^®^ or similar materials to avoid cross-contamination.
14. Proceed with the placement of TMJ prostheses, including the glenoid fossa and mandibular component.
15. Close the extraoral approaches.
16. Check final occlusion.

For unilateral TMJR with concomitant orthognathic surgery, it is essential to have two full sets of instruments to ensure efficiency and minimize the risk of cross-contamination. The recommended sequence of procedures for unilateral TMJR is as follows:

1. The patient’s face, neck, ears, nose, mouth and endotracheal tube are prepped with betadine and draped following standard protocols. Ear canals are packed with cotton pledges.
2. Isolate the mouth using appropriate barriers such as Ioban^®^, Tegaderm^®^ or similar materials to avoid cross-contamination.
3. Perform the unilateral extraoral approaches, including endaural/preauricular and retromandibular/submandibular incisions.
4. Perform condylectomy, discectomy, debridement and coronoidotomy/coronoidectomy as needed.
5. Isolate the TMJ surgical field using suitable barriers (Ioban^®^, Tegaderm^®^, etc.) to maintain sterility.
6. Placement of additional surgical drapes over the previous one to avoid cross-contamination.
7. Cut through the oral barrier membrane (e.g., Ioban^®^, Tegaderm^®^, etc.).
8. Perform the sagittal split osteotomy (SSO) on the contralateral side of the TMJR.
9. Place the intermediate splint and secure MMF.
10. Remove the additional surgical drapes. The surgeons change gloves and gowns.
11. Isolate the mouth using appropriate barriers such as Ioban^®^, Tegaderm^®^, or similar materials to maintain a sterile field.
12. Proceed with the placement of TMJ prosthesis, including the glenoid fossa and mandibular component.
13. Close the extraoral approaches.
14. Check stability of MMF and reinforce if necessary.
15. Stabilize the SSO with appropriate osteosynthesis materials.
16. Check obtained intermediate occlusion to ensure proper alignment and occlusion.
17. Complete the maxillary osteotomy and genioplasty as needed.
18. Check final occlusion.

The specific steps and techniques involved may vary depending on the type of TMJR prosthesis used and the individual patient’s needs. It is crucial to adhere to established surgical protocols and maintain a sterile environment throughout the procedure to minimize the risk of complications and promote successful outcomes.

## 7. Surgical Procedure

### 7.1. Intubation

Patients with advanced TMJ disease requiring TMJR often exhibit decreased MIO and commonly present with multiple risk factors for difficult intubation. To ensure successful intubation, a comprehensive preoperative clinical evaluation, thorough airway assessment, and effective communication with the anesthesiologist are crucial.

The authors strongly recommend employing an asleep fiberoptic nasal intubation technique performed by an anesthesiologist experienced in managing difficult airways. This approach allows for better visualization and maneuverability in challenging airway situations, increasing the likelihood of successful intubation. The use of fiberoptic technology provides enhanced precision and control during the intubation process. In some cases, the severity of the airway anatomy may necessitate awake fiberoptic intubation.

### 7.2. Patient Preparation

After nasotracheal intubation is completed, a 2.0 Silk suture is passed through the nasal septum behind the columella to secure the tube. A headwrap is placed and taped securely, and the endotracheal tube is secured to the headwrap with silk tape.

The patient is given intravenous antibiotics and corticosteroids. The patient’s face, neck, ears, nose, mouth and endotracheal tube are prepped with betadine and draped following standard protocols. Ear canals are packed with cotton pledges. As mentioned before, cross-contamination poses a significant risk of infection, which has been reported by multiple authors as the most frequent cause of failure, often necessitating replacement with a new prosthesis [26,27,28,29,30]. Due to the significant risk associated with contamination and subsequent infection, the authors recommend placing an iodine impregnated surgical incise drape (Ioban^®^) over the face and surgical sites with special care to cover the oral and nasal cavities. A sterile ear plug is then placed inside the ear canal.

### 7.3. Surgical Approaches

Multiple surgical approaches have been described for accessing the TMJ region during TMJR procedures. The commonly utilized incisions include the endaural, preauricular and rhytidectomy approaches, primarily employed for placing the glenoid fossa component. The most common complication associated with these approaches is injury to the facial nerve, the temporal branch is the most affected branch. There is a wide range of incidences of facial nerve injury associated with open TMJ surgery, ranging from 10 to 71% [31,32,33,34,35,36]. However, most of them are transient and typically resolved in three to six months.

**Figure 3:**
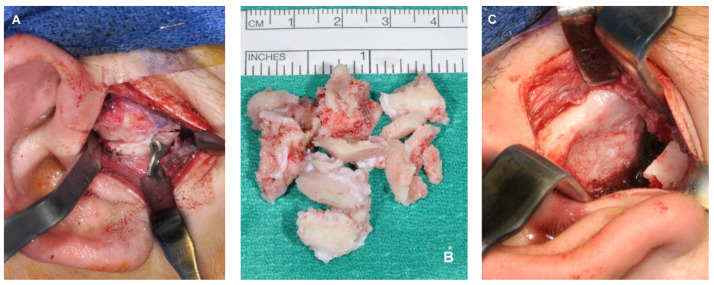
(**A**) Endaural approach for excision of TMJ ankylosis. (**B**) Bony segments resected from ankylosed TMJ. (**C**) Glenoid fossa following resection of TMJ ankylosis and recontouring of the glenoid fossa.

The authors prefer an endaural incision and consider it an optimal and safe approach that offers excellent cosmetic results and sufficient access to the TMJ, even in complex cases such as the removal of TMJ ankylosis (Figure 3). The detailed step-by-step procedure for this approach has been previously published [37], and to avoid redundancy, the specific information will not be reiterated here.

Typically, in order to access the ramus and facilitate the placement of the condylar component, an additional approach such as the submandibular or retromandibular incision is required. It is the authors’ preference to utilize a small submandibular approach positioned 1.5 to 2 cm below the angle of the mandible. This approach offers several advantages, including a safer dissection that remains posterior to the submandibular gland and anterior to the retromandibular vein. As a result, it obviates the necessity for dissection through the parotid gland, dissection around the submandibular gland, and minimizes the risk of encountering the marginal mandibular branch of the facial nerve. However, a nerve stimulator is still used during this dissection.

Recently, some authors have introduced minimally invasive approaches to perform TMJR using an endaural [38] or preauricular [39] approach only. It is important to note, however, that the complexity of the procedure should be taken into account. Consequently, the authors do not recommend the utilization of these minimally invasive techniques for cases that involve concomitant orthognathic surgery, due to the potential challenges they may present.

### 7.4. Condylectomy/Discectomy/Joint Debridement

**Figure 4:**
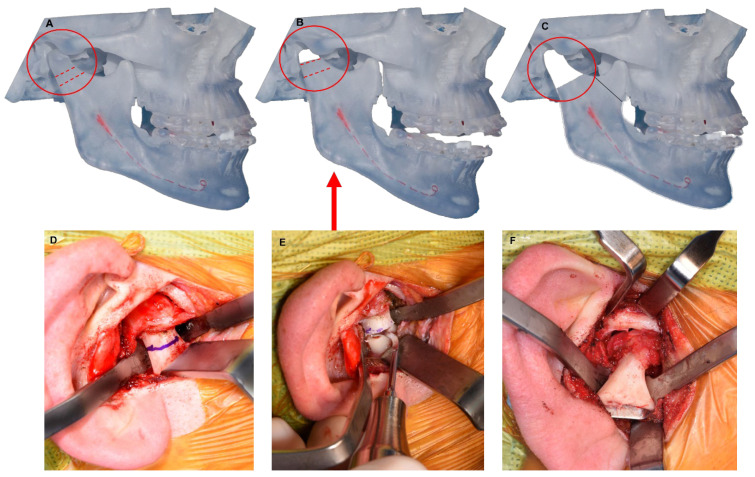
Two-step condylectomy. (**A**) Diagram showing two osteotomy sites for condylectomy. (**B**) Diagram showing the mandible pushed upward after initial condylectomy in order to facilitate access for the second osteotomy. (**C**) Diagram showing adequate space for the TMJR after condylectomy is performed. (**D**) Clinical photo showing special medial TMJ retractors in place and osteotomy design for condylectomy. (**E**) Piezo electric device used to perform condylectomy. (**F**) Condylar process is rotated outward to facilitate the release of the lateral pterygoid muscle insertion.

The authors strongly recommend a two-step osteotomy technique for condylectomy performed through the endaural incision using a piezoelectric device. After exposing the neck of the condyle, careful dissection is performed anterior and posterior to the neck of the condyle, Surgicel^®^ is packed medially and special medial TMJ retractors are placed. This helps protect the medial vessels and soft tissue structures while the osteotomy is performed. Once the neck of the condyle is isolated, an initial osteotomy is performed to separate the condyle. The condyle is then rotated outward to facilitate the release of the lateral pterygoid muscle insertion, this is conducted electrocautery to minimize bleeding. Following the removal of the condyle, the angle of the mandible is pushed up in order to perform the second osteotomy to allow for proper space for the TMJR (Figure 4). This approach offers notable benefits, including reducing the requirement for extensive inferior retraction of the soft tissues and minimizing the risk of facial nerve injury and bleeding as the facial nerve and maxillary artery branches are in close proximity.

**Figure 5:**
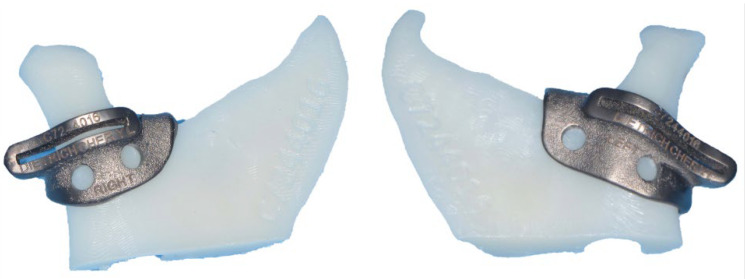
A. Metal cutting guides for condylectomy.

While virtual surgical planning (VSP) and cutting guides have been employed for condylectomy to ensure the osteotomy is performed at the planned level (Figure 5), the authors do not routinely utilize them.

After condylectomy is completed, a discectomy is performed. A hemostat forceps is utilized to gently pull the disc and an electrocautery is used to release the anterior attachment of the disc from the lateral pterygoid muscle. It is recommended to do this slowly in coagulation mode in order to control bleeding from the muscle. It is critical to make sure that the entire disc is removed since this can turn into heterotopic bone and affect the appropriate function of the joint or create a TMJ ankylosis.

Lastly, joint debridement is performed with curettes and occasionally with pineapple burs in order to remove all the fibrocartilage surrounding the TMJ since this is also a potential source of heterotopic bone formation.

### 7.5. Coronoidotomy/Coronoidectomy

Certain cases, such as those involving a history of ankylosis, significant mandibular advancement or counterclockwise rotation with a notable increase in posterior facial height, can benefit from the release of the coronoid process from the mandible. Generally, two options are available for this procedure:

- Coronoidotomy: This involves performing an osteotomy across the coronoid process, from the sigmoid notch to the anterior border of the ramus, allowing the coronoid process to be pulled superiorly by the temporalis muscle without complete removal.
- Coronoidectomy: This entails the complete removal of the entire coronoid process.

It is important to note that the authors recommend removing the coronoid process (coronoidectomy) solely in patients with TMJ ankylosis to minimize the risk of re-ankylosis. For other TMJ pathologies, the routine use of coronoidectomy is typically unnecessary and increases the likelihood of complications such as temporalis muscle fibrosis, pain and reduced MIO.

**Figure 6:**
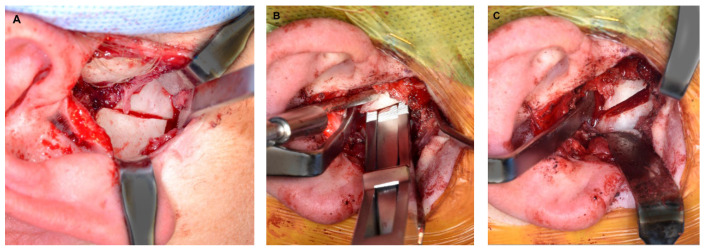
(**A**) Coronoid process is visualized after pushing mandible posteriorly and superiorly. Osteotomy is performed with a piezo electric device. (**B**) Smith bone spreader is used to separate the coronoid process. (**C**) Coronoid process is separated from the mandible.

After completing the condylectomy, the mandible can be pushed posteriorly and superiorly to enhance the visualization of the coronoid process. After the coronoid process is visualized, careful dissection is performed at the sigmoid notch and anterior border of the coronoid, Surgicel^®^ is packed medially and special medial TMJ retractors are placed. This helps protect the medial vessels and soft tissue structures while the osteotomy is performed. The authors perform the coronoidotomy or coronoidectomy using a piezo electric device through the endaural incision cutting from the anterior aspect of the coronoid process into the sigmoid notch. A Smith bone spreader is utilized to separate the coronoid process (Figure 6).

### 7.6. Flattening of the Ramus

**Figure 7:**
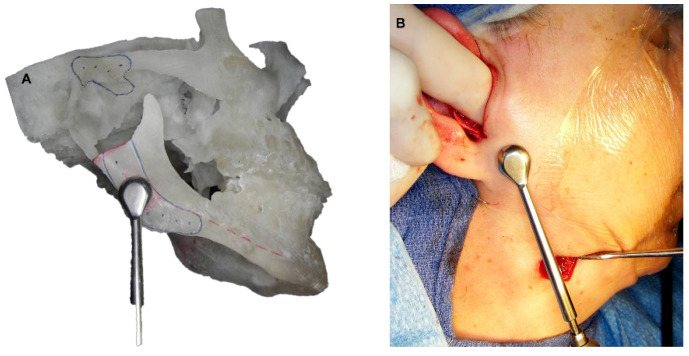
(**A**) STL model showing the use of reciprocating rasp used to flatten the mandibular ramus. (**B**) Clinical photo showing the reciprocating rasp that will be inserted into the submandibular incision to flatten the ramus.

A reciprocating rasp is used to flattening the mandibular ramus through the submandibular incision (Figure 7). The goal is to remove any interferences that were removed in the STL model during the fabrication of the prosthesis, this will improve adaptation of the prosthesis to the mandibular ramus. Additionally, it allows the surgeon to manipulate the mandibular component up-down in order to make sure that the mandibular component is fully seated in the glenoid fossa component. We prefer a flat mandibular component in all cases, allowing for some leeway to sit the joint passively in the most superior and posterior position.

### 7.7. Mobilization of the Mandible

**Figure 8:**
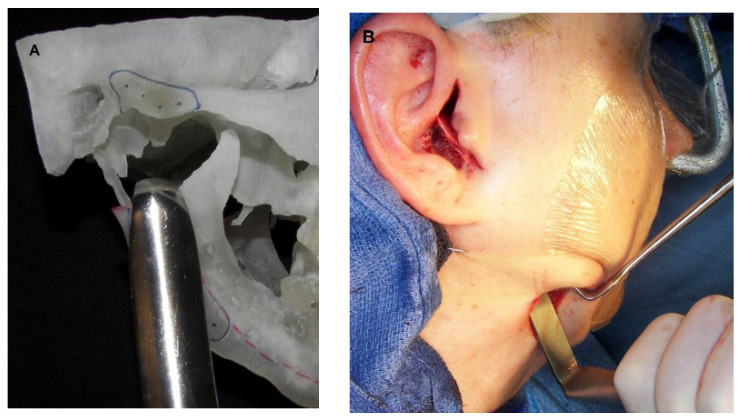
(**A**) Mandibular mobilizer device (KLS Martin, Jacksonville, FL, USA) hooked over the top of the sigmoid notch/condyle area. (**B**) Clinical photo showing the use of the mandibular mobilizer through the submandibular incision to mobilize the mandible anteriorly and inferiorly for mandibular advancements and counterclockwise rotations.

When mandibular advancement or counterclockwise rotation is planned, the mobilization of the mandible is a critical step to ensure that the mandible passively goes into its final position and fits into the intermediate splint. A mandibular mobilizer device (KLS Martin, Jacksonville, FL, USA) is inserted through the submandibular incision and hooked over the top of the sigmoid notch/condyle area and pulled downward and forward to facilitate vertical lengthening and advancement of the mandibular ramus (Figure 8). When performing substantial mandibular advancement and counterclockwise rotation, care must be taken to avoid intraoral tears or inferior alveolar nerve (IAN) avulsion. To minimize the risk of intraoral tears, in certain situations, the mucosa from the anterior border of the ramus can be carefully dissected via the submandibular approach.

### 7.8. Maxillomandibular Fixation

**Figure 9:**
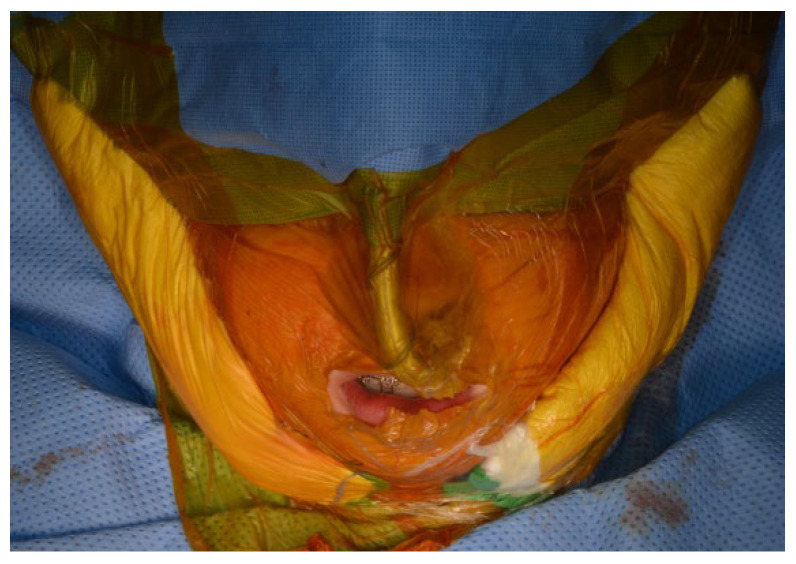
TMJ surgical field is isolated using Ioban^®^.

**Figure 10:**
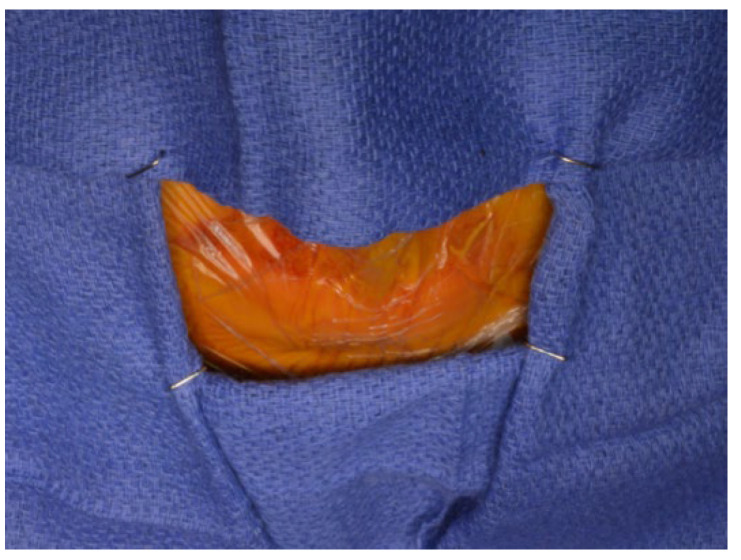
Additional surgical drapes are placed over previous ones and the exposure is limited to avoid cross-contamination.

After the mandible has been mobilized and the preparation of the TMJ area is completed, the TMJ surgical field is isolated using suitable barriers (Ioban^®^, Tegaderm^®^, etc.) to maintain sterility (Figure 9). Additional surgical drapes are placed over previous ones and the exposure is limited to avoid cross-contamination (Figure 10).

The Ioban^®^ barrier over the mouth is carefully cut through to expose the oral cavity. In cases where significant mandibular advancement or counterclockwise rotation is planned, there is an increased risk of mucosal tear along the anterior border of the mandible. Therefore, upon accessing the oral cavity, it is crucial to thoroughly examine for any perforations. If a perforation is detected, extensive irrigation with 0.12% Chlorhexidine is performed and the perforation is meticulously closed using a watertight closure technique with 3-0 Vicryl sutures.

Next, the intermediate splint is placed, and the patient is placed into MMF. For this step, the authors recommend placement of intermaxillary fixation (IMF) screws, even if the patient has orthodontic appliances since this helps to bring the mandible to the splint and provides a more stable MMF. Since the occlusion is not typically checked after the joint is placed, we rely on a very stable and firm MMF using multiple IMF screws and 24-gauge wire.

After this is completed, the additional surgical drapes are removed and the surgeon changes gloves and gown. The oral cavity is isolated again using appropriate barriers such as Ioban^®^, Tegaderm^®^ or similar materials to avoid cross-contamination.

### 7.9. Placement of the Glenoid Fossa Component

**Figure 11:**
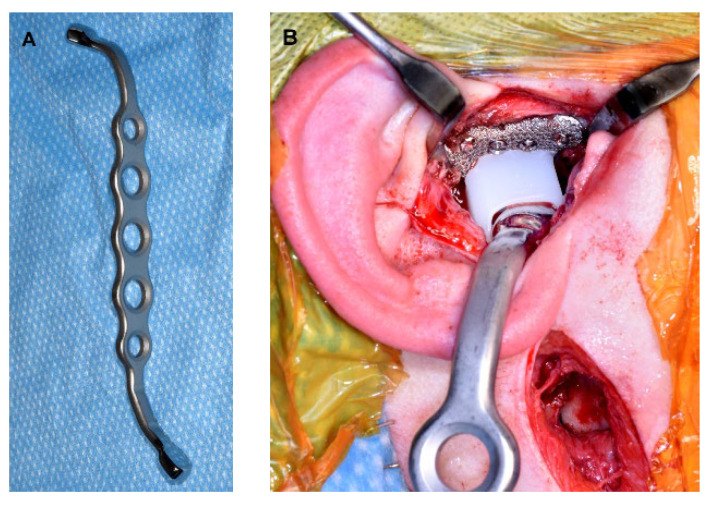
(**A**) Pusher device. (**B**) Pusher device is used to seat the glenoid fossa component into its final position.

The Ioban^®^ covering the TMJ surgical field is removed, and the area is irrigated profusely with normal saline and topical antibiotic. The authors do not recommend opening the sterile package of the prosthesis until when ready to place it. Once the surgical area is ready, the glenoid fossa component is placed, avoiding contact with the skin. A pusher device is used to put vertical pressure and to seat the glenoid fossa component into its final position and then the device is fixated to the zygomatic arch using monocortical screws using the device manufacturer’s recommendations for screw length (Figure 11).

### 7.10. Placement of the Mandibular Component

**Figure 12:**
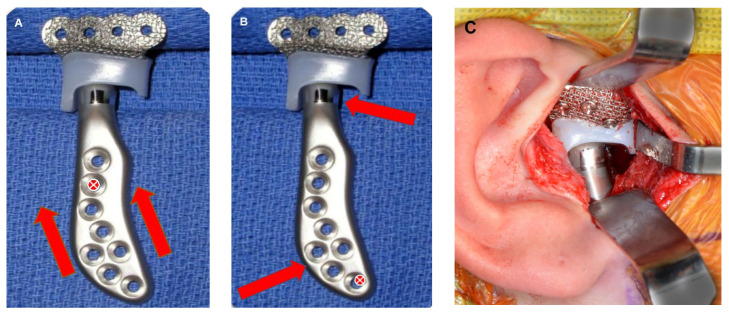
(**A**) Mandibular component is gently pushed superiorly and posteriorly to seat it into the glenoid fossa component. The first hole to be drilled should be one of the most superior holes (red cross). The hole is drilled in the uppermost portion, aiming superiorly and the first screw inserted but not tightened fully, this will push the mandibular component superiorly (red arrows). (**B**) The inferior portion of the mandibular component is pushed anteriorly. This rotation of the condylar prosthesis around the first screw positions the condylar head posteriorly into the fossa component (red arrows). The second hole to be drilled is the most inferior one (red cross), and it is drilled in the most anterior superior portion. (**C**) The condylar component is fully seated into the most superior and posterior zone of the glenoid fossa component.

The submandibular incision area is thoroughly irrigated with normal saline and topical antibiotic solution. It is important to note that the authors advise against opening the sterile package of the prosthesis until the surgical area is fully prepared. Once the surgical site is ready, the mandibular component is carefully placed, taking precautions to prevent direct contact between the device and the skin. Ensuring proper seating of the mandibular component into the superior and posterior region of the glenoid fossa component is crucial. To ensure proper placement and alignment of the mandibular component, the authors follow the following steps (Figure 12):

1. Using a Molt #9 periosteal elevator, the mandibular component is gently pushed superiorly and posteriorly to seat it into the glenoid fossa component.
2. The first hole to be drilled should be one of the most superior holes.
3. Carefully drill the hole in the uppermost portion, aiming upwards.
4. Insert a screw into the hole without fully tightening it down, this allows for adequate vertical placement of the mandibular component.
5. Proceed by pushing the inferior portion of the mandibular component forward. This positions the condylar prosthesis posteriorly into the fossa component, assuring a snug fit to maintain proper occlusion.
6. The second hole to be drilled is the most anterior one.
7. Drill the hole in the most anterior superior portion.
8. Fully tighten both screws securely.
9. Verify the position of the prosthesis and ensure that it is fully seated in the most superior and posterior zone of the glenoid fossa component.
10. If the prosthesis is in the correct position, continue drilling the remaining holes and place screws accordingly. A trocar could be used to drill and place the screws that are difficult to access from the submandibular incision.

When employing a stock prosthesis, it is crucial to follow the same sequence; however, utmost care must be taken to prevent posterior dislocation of the mandibular component due to the absence of a posterior stop in the stock fossa component.

After the devices are placed and before the incisions are closed, the surgical sites are thoroughly irrigated with normal saline and topical antibiotic solution. Additionally, the wounds are irrigated profusely with 0.05% chlorhexidine in a sterile water solution (Irrisept^®^).

### 7.11. Placement of Fat Graft or Other Materials

**Figure 13:**
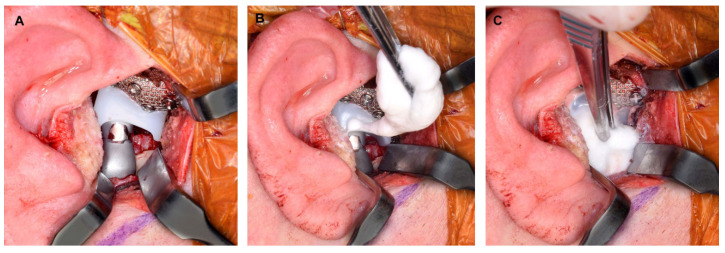
(**A**) Glenoid fossa and mandibular component in place. (**B**) Surgicel SNoW^®^ is packed to fill the dead-space surrounding the joint prosthesis. (**C**) Surgicel SNoW^®^ around TMJR.

The utilization of fat grafts harvested from the abdomen or groin has been widely reported as an excellent approach to fill the dead-space surrounding the joint prosthesis [40,41,42,43]. The authors frequently employ this technique in the majority of cases, although it does involve an additional procedure for fat harvesting. However, for patients who prefer to avoid an extra surgical procedure, the authors have been using oxidized regenerated cellulose (Surgicel SNoW^®^) mixed with antibiotics as an alternative method for the past five years (Figure 13). Notably, there have been no signs of heterotopic bone formation observed in these patients to date.

### 7.12. Closure of Incisions

There is a difference in preference among surgeons regarding the timing of checking the intermediate occlusion (for bimaxillary cases) or final occlusion (for mandibular-only cases) during the surgical procedure. While some surgeons prefer to evaluate the occlusion before closing the TMJ incisions, the authors’ preference is to close the TMJ incisions first in order to minimize the risk of cross-contamination. However, it is crucial to consider the surgeon’s experience and expertise when making this decision. If the obtained occlusion does not align with the planned occlusion, it may be necessary to re-open the surgical approaches and adjust the positioning of the prostheses accordingly.

The endaural incision is closed in layers using the standard fashion with 2-0, 3-0 Vicryl for the deep layers and 6-0 Prolene for the skin. Before closing the submandibular incision, the drill is used to make two or three bicortical holes on the mandibular angle and 2-0 Vicryl is used to reattach the masseter muscle to the mandible. After this, the submandibular incision is closed in layers in the standard fashion. Once surgical approaches are closed, the incisions are covered with Steri-Strips™ and the area is isolated again to proceed with the orthognathic component of the procedure.

### 7.13. Le Fort I osteotomy/Genioplasty

After completing the TMJR procedure, access to the oral cavity is gained, and the MMF is released. The intermediate splint is removed, and the occlusion is carefully evaluated to ensure proper alignment of the intermediate occlusion. Once the occlusion is confirmed to be appropriate, a conventional Le Fort osteotomy is performed and the patient is placed into MMF with the final occlusion. It is critical to remove all maxillary interferences to reduce the risk of displacing the mandibular components out of the fossa, which is very easy to do because of the laxity of the mandibular ramus from the TMJ prosthesis surgery.

**Figure 14:**
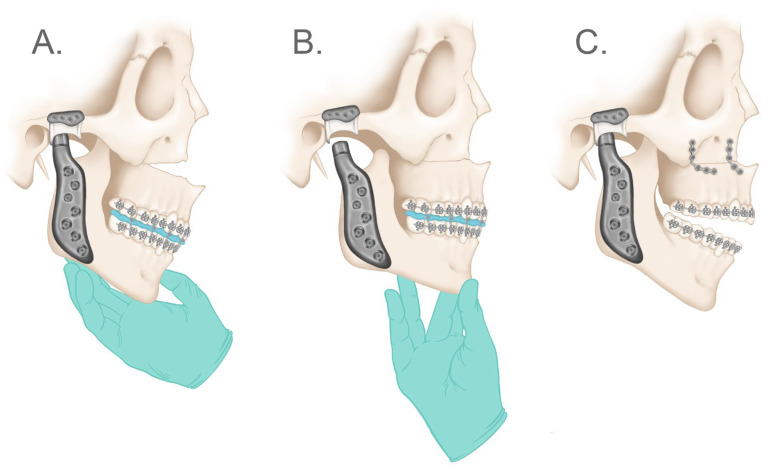
(**A**) Posterior maxillary interference. (**B**) Bilateral misplacement of the prosthesis outside the fossa when repositioning Le Fort osteotomy. (**C**) Anterior open bite noted after joint is carefully repositioned back into the fossa.

During the repositioning of the maxillary segment, it is of utmost importance to verify and ensure that the mandibular components of the TMJR are appropriately seated in the fossa. Neglecting this crucial step can result in fixing the Le Fort into the incorrect position. Bilateral misplacement of the prosthesis outside the fossa can lead to an anterior open bite (Figure 14), while if only one side is misaligned, it can cause a shift in the maxillary midline alignment once the mandibular component is correctly positioned back into place. In the event of any misalignment or improper positioning, the first step is to remove the maxillary hardware. The joint is carefully repositioned back into the fossa using bimanual manipulation, ensuring their appropriate seating. Once the mandibular components are correctly placed, the maxilla is re-stabilized to achieve the desired alignment.

Following this, the MMF is released, allowing for the verification of the final occlusion. After confirming the final occlusion, the maxillary incision is closed using standard surgical techniques.

If a genioplasty procedure was planned as part of the treatment, it is performed as the final step. Special attention and caution should be applied during a concomitant genioplasty procedure to minimize the risk of complications, particularly the risk of infection of the joint prosthesis. To mitigate this risk, a conservative posterior dissection technique along the symphysis/body region should be employed. This careful dissection helps prevent any communication with the site of the TMJR device.

### 7.14. Postoperative Management

Postsurgical vertical elastics are used to guide the occlusion and to support the mandible. Additionally, this helps to reduce the risk of dislocation of the prostheses. In our institution, patients are not kept in MMF. The elastics are removed the following morning and the patients are allowed to function right away.

Antibiotic therapy is prescribed (1st generation Cephalosporins) and wound care is recommended to keep the incisions clean and covered with antibiotic ointment for one week. Other postsurgical patient management is the same as routing bimaxillary orthognathic surgery including pain management, soft diet for eight weeks. A follow up appointment is scheduled one week after surgery, sutures are removed and active and passive range of motion exercises are explained. The initial goal of the physical therapy is 20 to 25 mm at 4 weeks, 30 to 35 mm at 8 weeks and over 35 mm at 12 weeks. If the patient has not met the goal at eight weeks, a formal physical therapy consult is initiated; however, this is rarely needed.

## 8. Conclusions

**Figure 15:**
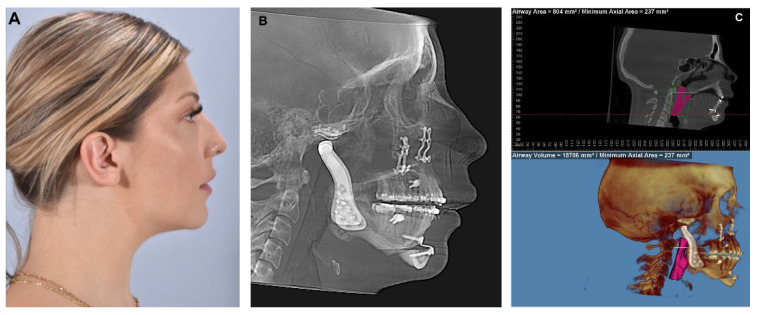
(**A**) The same patient showed previously. Patient was treated in a single surgical stage with bilateral TMJR, Le Fort I osteotomy advancement and genioplasty. The patient is seen 18 months post-surgery showing the maintenance of good facial balance. (**B**) Lateral cephalogram showing adequate facial profile, correction of the occlusal plane angle, hardware in place with no signs of complications. (**C**) Airway analysis shows significant improvement in airway space (Airway volume = 18,786 mm^3^, Minimum Axial Area = 237 mm^2^).

In conclusion, the concomitant approach of TMJR and orthognathic surgery presents a viable treatment option for individuals with complex craniofacial conditions. This combined procedure aims to address both functional and aesthetic concerns (Figure 15), offering a comprehensive solution for patients suffering from TMJ disorders and skeletal malocclusions and does so in one surgical procedure.

However, it is crucial to consider the complexity and potential risks associated with the concomitant procedure. The surgeon’s experience, careful patient selection, thorough preoperative planning and close postoperative monitoring are essential for minimizing complications and optimizing patient outcomes.

## Data Availability

Data sharing is not applicable to this article.
